# Successful treatment of thyrotoxicosis is accompanied by a decrease in serum sclerostin levels

**DOI:** 10.1186/1756-6614-5-14

**Published:** 2012-11-13

**Authors:** Elżbieta Skowrońska-Jóźwiak, Kinga Krawczyk-Rusiecka, Krzysztof C Lewandowski, Zbigniew Adamczewski, Andrzej Lewiński

**Affiliations:** 1 Department of Endocrinology and Metabolic Diseases, Polish Mother’s Memorial Hospital - Research Institute, Rzgowska St. No. 281/289, 93-338, Lodz, Poland; 2 Medical University of Lodz, Lodz, Poland

## Abstract

**Abstract:**

Sclerostin, a product of a *SOST* gene, is a protein expressed by osteocytes that inhibits osteoblastic bone formation. Several hormones, including PTH and glucocorticosteroids, have been suggested to be possible regulators of sclerostin production. The influence of thyroid hormones on sclerostin synthesis has not been investigated, so far. The aim of the study was to evaluate sclerostin concentrations in patients before and after treatment of thyrotoxicosis.

**Patients and methods:**

The study involved 15 patients (4 men), mean age 51.8±15.3 years, mean BMI value - 24.7±3.5, with thyrotoxicosis due to Graves’ disease or toxic multinodular goitre. Serum sclerostin was measured by immunoassay at diagnosis of thyrotoxicosis and after 6–10 weeks of treatment with thiamazole. The data were analysed by means of simple descriptive statistics of location and dispersion and Mann–Whitney *U* test for pairs of results, before and after thiamazole therapy. Association between variables was evaluated with use of Spearman`s correlation coefficient.

**Results:**

There was a significant decrease in free T3 (FT_3_) and free T4 (FT_4_) concentrations (from 8.74±4.79 pg/ml to 3.54±2.40 pg/ml, and from 4.48±2.21 ng/ml to 1.02±1.07 ng/ml, respectively, p<0.001). This was accompanied by a marked decrease of serum sclerostin levels from 55.46±20.90 pmol/l to 35.73±15.70 pmol/l, p<0.0015). Interestingly, enough, sclerostin levels did not correlate with serum FT_3_ or FT_4_ concentrations.

**Conclusions:**

Restoration of a euthyroid state in patients with thyrotoxicosis results in a significant decrease in serum sclerostin concentrations. The above mentioned phenomenon may reflect lowering of bone metabolism, but a possible direct influence of thyroid hormones on *SOST* gene needs to be investigated.

## Background

Sclerostin, a product of a *SOST* gene, is a protein expressed by osteocytes and inhibits osteoblastic bone formation. Sclerostin deficiency has been implicated in the pathogenesis of bone sclerosing dysplasias, i.e. sclerosteosis and van Buchem disease [1]. Several hormones have been suggested to serve as possible regulators of sclerostin production. PTH decreased sclerostin production *in vitro* in mice [2], as well as *in vivo* in humans [3]. On the other hand, calcitriol alone or in combination with retinoic acid increased *SOST* expression in human osteoblastic cells *in vitro*[4]. Glucocorticoids are thought to stimulate sclerostin secretion from osteocytes [5].

The influence of thyroid hormones on sclerostin synthesis has not been investigated so far, however the influence of hypothalamus-pituitary-thyroid axis on bone metabolism is well known [6]. Thyroid hormones are necessary for normal growth. Population studies indicate that both hypothyroidism and hyperthyroidism are associated with an increased risk of fractures [7] .

The aim of the study was to evaluate sclerostin concentrations in patients before and after treatment of thyrotoxicosis.

## Patients and methods

The study was approved by the Ethics Committee of the Polish Mother's Memorial Hospital - Research Institute in Lodz, Poland. The study involved 15 patients (4 men), mean age 51.8±15.3 years, mean value of BMI - 24.7±3.5 kg/m^2^, with thyrotoxicosis due to Graves’ disease or toxic multinodular goitre. Characteristics of examined population before and after treatment was shown in Table 1. Serum sclerostin was measured by with a quantitative sandwich ELISA by Biomedica (Vienna, Austria) at diagnosis of thyrotoxicosis and after 6–10 weeks of treatment with thiamazole. The intraassay coefficient of variance (CV) is 5%, and the interassay coefficient of variance (CV) is 3–6%. The data were analysed by means of simple descriptive statistics of location and dispersion and Mann–Whitney`s *U* test for pairs of results before and after thiamazole therapy. Association between variables was evaluated with use of Spearman`s correlation coefficient.

**Table 1:** Descriptive statistics for demographic characteristics of the tested sample (n=15)

	**Mean**	**Median**	**SD**	**Min**	**Max**	**p-value**
Age [years]	51.8	59.0	15.3	18.0	69.0	
Body mass - before [kg]	69.3	66.0	12.7	48.0	98.0	0.051
Body mass - after [kg]	70.5	72.0	12.1	51.0	98.0	
BMI value - before [kg/m^2^]	24.7	23.8	3.5	1.5	1.9	0.033
BMI value - after [kg/m^2^]	25.2	25.4	3.4	19.6	31.7	
Heigth	1.7	1.6	0.1	1.55	1.82	-

## Results

There was a significant decrease in FT3 and FT4 concentrations (from 8.74±4.79 pg/ml to 3.54±2.40 pg/ml, and from 4.48±2.21 ng/ml to 1.02±1.07 ng/ml, respectively p<0.001). This was accompanied by a marked decrease of serum sclerostin levels from 55.46±20.90 pmol/l to 35.73±15.70 pmol/l, p<0.0015) (Table 2). Interestingly, enough, neither sclerostin nor TSH levels did not correlate with serum FT3 or FT4 (Tables 3 and 4).

**Table 2:** **Descriptive statistics for the measurement results *before *and *after *thiamazole therapy (not all subjects have both measurements)**

	**Mean**	**Median**	**SD**	**Min**	**Max**	**p-value**
anti-TPO *– before* [IU/ml]	222.7	21.8	356.0	5	1000	*-*
anti-Tg *– before* [IU/ml]	109.2	20.2	222.7	10	803	*-*
anti-TSHR – *before* [IU/l]	7.6	1.3	11.6	0.3	40.0	*0.999*
anti-TSHR – *after* [IU/l]	11.9	10.9	5.9	6.6	18.3	
TSH *- before* [mIU/l]	0.0	0.0	0.0	0.0	0.0	*0.013*
TSH *– after* [mIU/l]	1.1	0.0	1.7	0.0	5.8	
FT3 *– before* [pg/ml]	8.74	7.53	4.79	4.02	16.5	*0.0010*
FT3 *– after* [pg/ml]	3.49	3.54	2.40	0.74	4.8	
FT4 *– before* [ng/ml]	4.48	2.21	1.01	7.84	32.5	*0.0010*
FT4 *– after* [ng/ml]	1.02	1.07	0.44	0.34	1.7	
Sclerostin*- before* [pmol/l]	55.46	41.90	20.90	39.29	148.8	*0.0015*
Sclerostin *– after* [pmol/l]	35.73	27.20	15.70	23.46	99.6	

P-value for the Mann–Whitney`s *U* test for a comparison of pairs of measurement results *before* and *after* antithyroid therapy (where appropriate).

**Table 3:** **Spearman rang correlation coefficients (r_s_) for FT3 (*before *and *after*) and other measurement results**

	**FT3 *- before***		**FT3 *- after***	
	**r**_**s**_	**p-value**	**r**_**s**_	**p-value**
TSH	0.535	0.040	−0.464	0.094
Sclerostin	0.079	0.781	0.064	0.829

**Table 4:** **Spearman rang correlation coefficients (r_s_) for FT4 (*before *and *after*) and other measurement results**

	**FT4 *- before***		**FT4 *- after***	
	**r**_**s**_	**p-value**	**r**_**s**_	**p-value**
TSH	0.252	0.365	−0.489	0.076
Sclerostin	−0.004	0.990	0.240	0.409

## Discussion

We demonstrated high levels of sclerostin in thyrotoxicosis and a significant decrease of its serum concentrations coinciding with restoration of an euthyroid state. Pretreatment sclerosin levels in our study were higher than reference values presented by Biomedica (mean concentration - 19.3 pmol/l, range from 10.9 pmol/l to 28.7 pmol/l in serum), and by other authors - 33–37 pmol/l for healthy premenopausal women [6,7] and 49.8 pmol/l - for men [7].

Thyrotoxicosis is a common endocrine disorder, affecting several systems and organs including skeletal system. Thyrotoxicosis increases bone metabolism and leads to development of osteoporosis [8-10]. Hyperthyroid patients have high bone turnover and negative calcium and phosphorus balance [11]. These detrimental effects of thyrotoxicosis on bone metabolism are probably caused by overproduction of T3, mediated principally by T3 receptor alpha [12] or low TSH level [13,14].

There are several examples of negative impact of thyrototoxicosis on bones even, in subclinical hyperthyroidism, for instance low-normal TSH values are associated with high prevalence of vertebral fractures in women with post-menopausal osteoporosis or osteopenia, independently of thyroid hormones, age and BMD value [13]. This is one of the reasons why in subclinical hyperthyroidism treatment should be considered in the presence of risk factors, including osteoporosis [15].

Influence of thyrotoxicosis on bone seems to be temporary. Fracture risk was only significantly increased around the time of diagnosis but decreased to normal levels after restoration of an euthyroid state [9,10]. Furthermore, normalization of BMD, bone markers and osteoprotegerin was observed during treatment [10,11,16], however, some authors showed elevation of bone formation markers despite normalization of thyroid hormones and TSH levels [10,17]. We speculate that a decrease of sclerostin levels – as observed by us - may be associated with normalization of bone metabolism. It may also be argued that the above mentioned decrease in sclerostin levels in patients recovering from thyrotoxicosis might be mediated by PTH. This is because a fall of PTH concentration was reported in subjects with thyrotoxicosis [10,17], followed by an increase during anti-thyroid treatment [11]. There are also recent data demonstrating a decline of sclerostin concentrations by PTH. For instance, lowering of sclerostin levels was demonstrated after injection of PTH [3] and in patients with hyperparathyroidism [18,19]. Supposing the normalization of thyroid hormones is accompanied by an increase of PTH, one might argue that PTH *per se* might be responsible for the decline of sclerostin concentrations observed, in our study.

To the best of our knowledge, this study is the first in which a clear decrease in sclerostin concentrations resulting presumably from a decrease of thyroid function has been demonstrated. The cause of this phenomenon is, however, complex and requires further study.

## Competing interests

The authors declare that they have no competing interests.

## Authors’ contributions

ES-J designed the study, qualified the patients and prepared the draft of manuscript. KK-R and ZA qualified the patients and participated in data acquisition and in performing hormonal studies. KCL participated in acquisition of data and the scientific revision of the manuscript. AL, the senior author, wrote the final version of manuscript. All authors read and approved the final manuscript.
